# Midwives’ experience of their education, knowledge and practice around immersion in water for labour or birth

**DOI:** 10.1186/s12884-018-1823-0

**Published:** 2018-06-19

**Authors:** Lucy Lewis, Yvonne L. Hauck, Janice Butt, Chloe Western, Helen Overing, Corrinne Poletti, Jessica Priest, Dawn Hudd, Brooke Thomson

**Affiliations:** 1 School of Nursing, Midwifery and Paramedicine, Curtin University, Bentley, Perth, Western Australia 6102 Australia; 2 Department of Nursing and Midwifery Education and Research, King Edward Memorial Hospital, Subiaco, Western Australia 6008 Australia; 3 Family Birth Centre, Midwifery Group Practice and Community Midwifery Program, King Edward Memorial Hospital, Subiaco, 6008 Western Australia Australia

**Keywords:** Waterbirth, Mixed methods, Clinical confidence, Practice guideline, Learning, Education

## Abstract

**Background:**

There is limited research examining midwives' education, knowledge and practice around immersion in water for labour or birth. Our aim was to address this gap in evidence and build knowledge around this important topic.

**Methods:**

This mixed method study was performed in two phases, between August and December 2016, in the birth centre of a tertiary public maternity hospital in Western Australia. Phase one utilised a cross sectional design to examine perceptions of education, knowledge and practice around immersion in water for labour or birth through a questionnaire. Phase two employed a qualitative descriptive design and focus groups to explore what midwives enjoyed about caring for women who labour or birth in water and the challenges midwives experienced with waterbirth. Frequency distributions were employed for quantitative data. Thematic analysis was undertaken to extract common themes from focus group transcripts.

**Results:**

The majority (85%; 29 of 34) of midwives surveyed returned a questionnaire. Results from phase one confirmed that following training, 93% (27 of 29) of midwives felt equipped to facilitate waterbirth and the mean waterbirths required to facilitate confidence was seven. Midwives were confident caring for women in water during the first, second and third stage of labour and enjoyed facilitating water immersion for labour and birth. Finally, responses to labour and birth scenarios indicated midwives were practicing according to state-wide clinical guidance.

Phase two included two focus groups of seven and five midwives. Exploration of what midwives enjoyed about caring for women who used water immersion revealed three themes: instinctive birthing; woman-centred atmosphere; and undisturbed space. Exploration of the challenges experienced with waterbirth revealed two themes: learning through reflection and facilities required to support waterbirth.

**Conclusions:**

This research contributes to the growing knowledge base examining midwives' education, knowledge and practice around immersion in water for labour or birth. It also highlights the importance of exploring what immersion in water for labour and birth offers midwives, as this research suggests they are integral to sustaining waterbirth as an option for low risk women.

**Electronic supplementary material:**

The online version of this article (10.1186/s12884-018-1823-0) contains supplementary material, which is available to authorized users.

## Background

The provision of water immersion for labour and birth is facilitated by midwives working within low risk midwifery-led models of care who are deemed competent to provide this method of birth [1, 2]. The concept of competence is often aligned with confidence [3], but distinguishing between these two concepts is important as they are not always synonymous. A midwife may be a competent waterbirth practitioner having met all the professional competency requirements, but becoming confident is an individual journey that is dependent upon trust in clinical guidelines, presence of peer support and the challenge of achieving consistent exposure to waterbirth [4]. Additionally, midwives with extensive experience of conventional birth on land may be challenged to unlearn old skills and develop new practices required for water immersion in labour and birth. Whilst midwives working within low risk continuity of care models where physiological birth was the norm, researchers concluded that a supportive culture assisted in the development of their confidence, irrespective of clinical experience [4].

Individual midwives can act as gate keepers to water immersion which is more likely to be accepted into an organisation’s culture when it is supported by midwifery managers and championed by experienced waterbirth practitioners [5]. These champions can mentor midwives who wish to achieve waterbirth competency [5]. In this situation, mentors may not always be the most senior midwives who have extensive experience with conventional birth on land. Caution is recommended to recognise and consider ways to minimise the possible hierarchical tensions that may occur when experienced midwives are mentored by junior midwives who have achieved waterbirth competency [4]. Indeed, promoting and sustaining change in midwives’ waterbirth practice can be challenging. A study, undertaken in the United Kingdom (UK), utilised problem solving workshops to identify interventions that could develop and sustain a waterbirth culture. These interventions included: publishing monthly waterbirth statistics; setting a target of 100 waterbirths per annum; keeping portable birthing pools partially inflated; and appointing a waterbirth champion. Co-ordinators were able to positively influence midwifery practice through social support which was found to be pivotal in relation to developing and sustaining a waterbirth culture [6].

Access to immersion in water for labour and birth is reliant on both the care provider and the policies and procedures that guide clinical practice. Policies and guidelines in relation to water immersion for birth in Australia usually reflect the organisation’s interpretation of the current literature [7]. Additionally, more evidence is required around the effect of immersion in water on neonatal morbidity [1, 8, 9] and management of the third stage of labour [7]. A literature review exploring midwives concerns around waterbirth [10] identified three clinical issues (neonatal water aspiration and neonatal and maternal infection and thermo-regulation) and two practice issues (midwives skills and education and emergency procedures around maternal collapse). The clinical issues were not evidence based and the practice issues could ‘be addressed by appropriate policy, guidelines and practice’ [10]. Other work exploring how a convenience sample of 249 Australian midwives utilised normal birth guidelines, found that although the majority (90%) were aware that specific guidelines existed, only 71% reported routinely using them to guide their clinical practice [11].

It has been suggested that the waterbirth environment nurtures woman-centred care by facilitating shared decision making and perceptions of control around their care [8]. However, recent Australian research found some midwives perceive waterbirth policies and guidelines can limit their scope to facilitate water immersion and did not always support women’s informed choice [12].

There is limited research examining midwives' education, knowledge and practice around immersion in water for labour or birth. To address this gap in evidence and build our knowledge around this topic, our intention was to obtain a contemporary overview of midwives' experience of their education, knowledge and practice around immersion in water for labour or birth in Western Australia (WA).

## Methods

The specific aim of this WA study was to assess Midwifery Group Practice (MGP) and Community Midwifery Program (CMP) midwives’ experience of their education, knowledge and practice around immersion in water for labour or birth. This mixed method study was performed in two sequential phases. Phase one incorporated a cross sectional design and examined midwives' perceptions of education, knowledge and practice around immersion in water for labour or birth through a questionnaire; 34 midwives were invited to participate. Phase two employed a qualitative descriptive design to explore what midwives enjoyed about caring for women who labour or birth in water and the challenges midwives experienced with waterbirth; two focus groups were held.

### Design

Mixed methods were utilised to provide in-depth knowledge [13, 14] relating to the education, knowledge and practice around immersion in water for labour or birth. This methodology offers researchers using quantitative methods the opportunity to utilise qualitative research to gain deeper understanding of the investigated phenomenon [15]. Utilising this two phase mixed methodology provided a more informative, constructive and thorough integration of the research results, building on the links between methods rather than within methods [15]. We envisaged being able to utilise both numbers and words would give greater insight into the bigger picture around midwives' experience of their education, knowledge and practice around immersion in water for labour or birth.

### Participants and setting

The study was performed at the sole tertiary public maternity hospital in WA, which has approximately 5200 births annually. Women can labour and birth in the tertiary maternity hospital’s Labour Ward and Birth Suite or the Family Birth Centre (an adjacent building within the hospital grounds).

Perinatal data collected in 2016, by King Edward Memorial Hospital (KEMH) in WA confirmed that 5% (228 of 4402) of infants ≥37 weeks gestation were born immersed in water. Currently WA and South Australia are the only Australian states with state-wide policies and guidance supporting immersion in water for labour and birth, although waterbirth is available in every state and territory [16, 17]. In WA midwives are guided by state-wide clinical waterbirth guidelines [16]. Between August and November 2016 we invited the 34 midwives who provided care for women who opted to use water for labour and/or birth to participate. Throughout the study, women choosing to labour and/or birth in water were cared for by midwives working within two publically funded services: the MGP and CMP. These low risk continuity of care models [18] are ideally suited to provide care for women who labour and/or birth in water, as this model facilitates a shift from high risk obstetric-led care to low risk midwifery-led care [18, 19]. Both the MGP and CMP operate their services (antenatal, intrapartum and postnatal care) from the Family Birth Centre (FBC) with the CMP also providing antenatal, intrapartum and postnatal care to women in their homes and local community clinics. In these midwifery care models, a primary midwife is supported by a small team of midwives who provide continuity of care 24 h a day throughout pregnancy, birth and up to two weeks post birth. Perinatal data collected in 2016 at KEMH confirmed MGP and CMP midwives birthed 16% (813 of 5189) of all women at KEMH. Although, no women received immersion in water for labour and birth in the tertiary maternity hospital’s Labour Ward and Birth Suite throughout the duration of the study, in the last two weeks of the study the tertiary maternity hospital agreed that immersion in water for labour and birth could be facilitated in their main Labour and Birth Suite.

### Recruitment and data collection

#### Phase one

Midwives were invited to participate in the study through an information letter and in-house designed questionnaire (Additional file 1), both of which were sent to their workplace mobile phone. Midwives who did not want to complete the online questionnaire were given the option to complete a hard copy and return it to the research team by placing it in a locked box situated in the FBC. Returning a completed questionnaire was deemed implied consent. Ethics approval was gained from the Women and Newborn Health Service Ethics Committee (Approval Number 2016103QK) at the study centre.

The questionnaire was validated through a review process with an expert panel involving a midwifery educator and three midwives who had experience caring for women who had birthed in water. Feedback from the panel resulted in changes to questions around being competent to facilitate water immersion for labour or birth and actively promoting this birth choice for labour and birth. This question was divided into two questions, one focused upon labour and another concerning birth.

The aim of the questionnaire was to examine midwives' perceptions of education, knowledge and practice around immersion in water for labour. Midwives were asked about: their employment status (if they worked in the MGP or CMP and how long they had been working as a midwife and facilitating water immersion for labour or birth); their education (training undertaken to facilitate immersion in water for labour or birth and number of births required to develop waterbirth confidence); their practice (two factors they would discuss with women in relation to water immersion for labour or birth); their confidence caring for women immersed in water for labour and birth (in the first, second and third stages of labour); their enjoyment facilitating immersion in water for labour and birth; whether they actively promote water immersion for labour and birth; and their interpretation of four scenarios around antenatal, early labour, birth and third stage clinical care. The scenarios required a written response, were scored and were based on information relating to the state-wide clinical waterbirth guidelines [16]. It was decided to give midwives completing the questionnaire a website link to the state-wide guidelines [16], in the information letter accompanying the questionnaire. By providing a website link to this guidance, we were examining how midwives interpreted and applied the guidance in their clinical practice. In relation to confidence and enjoyment, midwives were asked to place a cross on a 10 cm line (where zero was ‘not confident’ or ‘does not enjoy’ and 10 was ‘very confident’ or ‘enjoys’), to quantify their perceptions on the continuum from zero to ten.

#### Phase two

An item was included at the end of the questionnaire (phase one) inviting midwives to participate in a focus group to discuss their experiences around immersion in water for labour or birth. The first author conducted the two focus groups. Observations were documented by the fourth author in the form of field notes. Each focus group lasted approximately 45 min. The focus groups were held at the study centre in an interview room that was convenient to all interested midwives. Prior to commencing the focus group, midwives were reminded that their privacy would be maintained by issuing each of them a unique identifier; the discussions linked to an individual’s identity should ‘remain in the room’; and that the focus group would be audio recorded. All midwives verbally consented to these conditions.

The final questions for the focus groups (Additional file 2) were based around the results from phase one, with two questions being developed: question one asked ‘What contributes to your enjoyment of waterbirth?’ Two prompts were utilised for this question. The first one addressed the promotion of natural birth and the second was around supporting women’s choice. Question two asked ‘Are there any issues with waterbirth?’ One prompt was utilised around the issue of exploring which stage of labour midwives found most challenging.

### Data analysis

#### Phase one: Quantitative data

Each of the four clinical scenarios was allocated a maximum score according to whether a midwife correctly identified key aspects of clinical practice based on the state-wide clinical waterbirth guidelines [16]. Four members of the research team independently scored each scenario. The team then met to compare scores. Any disagreement in relation to the scores was discussed and a consensus reached by referring back to the data.

Means, and interquartile ranges were used to summarise continuous data (such as the scores for each scenario). Frequency distributions were used to summarise categorical data (such as feeling equipped to facilitate waterbirth following training). Statistical software (SPSS version 22) was used for analysis.

#### Phase two: Qualitative data

Transcribed focus groups were subjected to thematic analysis [20] by five members of the research team, who analysed a cross-section of transcripts and field notes ensuring each data source was reviewed by at least two members [21]. Analysis required the research team to become submerged in the data. Transcripts and field notes were deconstructed enabling the research team to identify patterns, similarities and themes from the midwives’ words or sentences [13, 20, 21]. The team met weekly over three months to negotiate, clarify and refine the themes. Any disagreements on interpretation were negotiated by referring back to the data. All the researchers were clinical or academic midwives, with varying experiences of facilitating immersion in water for labour or birth. As a process of member checking, preliminary themes were presented to five midwife participants who confirmed agreement with the themes.

## Results

### Phase one

Table 1 summarises the midwives’ perception of their education, knowledge and practice around immersion in water for labour and birth. A total of 29 (85%) out of a potential 34 midwives returned a questionnaire. The mean time midwives were qualified was 162 months (13 years and 5 months), with the mean time midwives had been facilitating waterbirth being 83 months (eight years and 9 months). Most (59%; *n* = 17) midwives worked in the MGP. The majority (93%; *n* = 27) of midwives used the WA state-wide clinical guidelines for waterbirth [16] for their education and training, with 90% (*n* = 26) accessing the E-learning package developed by the study hospital’s education department. Following waterbirth training, 93% (*n* = 27) felt equipped to facilitate waterbirth with the mean number of waterbirths required to facilitate confidence being seven.

On a scale of 0 to 10 (where zero was ‘not confident’ and 10 was ‘very confident’), midwives were very confident caring for women in water during the first stage of labour (mean score of 10). They were also confident caring for women in the second stage (mean score of 9) and third stage of labour (mean score of 8). The mean score in relation to confidence using the emergency evacuation to get the woman out of the bath was eight. On a scale of 0 to 10 (where zero was ‘does not enjoy’ and 10 was ‘enjoys’), midwives enjoyed facilitating immersion in water and birth, obtaining a mean score of 10. Finally, mean scores for the antenatal, early labour, birth and third stage of labour scenarios indicated midwives were practicing according to the WA state-wide clinical guidelines for waterbirth [16].

**Table 1:** Midwives’ perception of their education, knowledge and practice around immersion in water for labour and birth

Outcomes	n=29 (n%)
Months qualified as midwife^a^	162(64-247)[10-454]
Months been facilitating water immersion for labour or birth^a^	83(36-120)[4-286]
Where do you work?	
Community Midwifery Program	12(41)
Midwifery Group Practice	17 (59)
Waterbirth training	
West Australian guidelines	27(93)
Hospital based E-learning	26(90)
Observed waterbirth by midwife competent in waterbirth	26(90)
Waterbirth supervised by midwife competent in waterbirth	25(86)
Hospital study day	15(52)
Following training felt well equipped to facilitate waterbirth	27(93)
Number of waterbirths required to facilitate confidence^a^	7(4-10)[0-30]
Primary factor discussed with women	
Safety	9(31)
Eligibility	8(28)
Benefits of waterbirth	7(24)
Waterbirth process	5(17)
Confidence caring for women in water during 1^st^ stage labour^a,b^	10(10-10)[8-10]
Confidence caring for women in water during 2^nd^ stage labour^a,b^	9(9-10)[6-10]
Confidence caring for women in water during 3^rd^ stage labour^a,b^	8(6-10)[2-10]
Confidence using emergency evacuation system^a,b^	8(8-10)[3-10]
Enjoy facilitating immersion in water for labour^a,c^	10(10-10)[8-10]
Enjoy facilitating immersion in water for birth^a,c^	10(10-10)[8-10]
Actively promote use of water for labour for all eligible women	28(97)
Actively promote use of water for birth for all eligible women	26(90)
Scenario 1 (Antenatal); Maximum 2 points^a^	2(1-2)[0-2]
Scenario 2 (Early labour); Maximum 3 points^a^	2(2-3)[0-3]
Scenario 3 (Birth); Maximum 2 points^a^	2(1-2)[0-2]
Scenario 4 (Third stage); Maximum 3 points^a^	2(2-3)[0-3]

^a^Mean (interquartile range)[range]

^b^Scale of 0-10. Where 0 ‘Not confident’ and 10 ‘Very confident’

^c^Scale of 0-10. Where 0 ‘Do not enjoy’ and 10 ‘Enjoy’

### Phase two

Two focus groups comprising of seven and five midwives were performed. Findings are presented with supportive quotes in italics from the midwives. For confidentiality a pseudo-name was allocated to each midwife.

#### Caring for women who labour or birth in water

Exploration of what midwives enjoyed about caring for women who labour or birth in water revealed three distinctive themes: instinctive birthing; woman-centred atmosphere; and undisturbed space (Table 2).

**Table 2:** What midwives enjoy about caring for women who labour or birth in water

Theme	Definition of Theme
Instinctive birthing	Nurtures instinctive birthing behaviour led by the woman
Woman-centred atmosphere	Provides an environment which is woman centred, calm and peaceful and relaxed.
Undisturbed space	Creates an undisturbed space where access is mediated by the water.

### Instinctive birthing

The theme ‘instinctive birthing’ described how midwives perceived labouring or birthing in water nurtured an instinctive birthing behaviour led by the woman. Anna reflected ‘*You absolutely see the hormones that promote labour take over. You know labour progresses better and the woman relaxes into labour*’. Noreen agreed; they ‘*Really feel what the body is able to do and how birth feels*’, whilst Kate described how she perceived water enabled her to trust a woman’s ability to instinctively birth:

*I think they progress really well. I don’t do many vaginal exams, but they are getting in* [the water] *and they are well established, they are fully before you know it and they don’t push early. Like sometimes with their first grunt the heads on view…They’re not asking for epidurals, they’re not asking for gas.*

Jasmine agreed with Kate’s sentiments: ‘*Because you can’t see as the vagina is submerged, the first sign she needs to push is she’s pushing*’ whilst Anna summarised her experience was that ‘*They’re more likely to reach down and lift the baby up themselves*’.

### Woman-centred atmosphere

The theme ‘woman-centred atmosphere’ described a labour and birth environment which was woman centred, calm, peaceful and relaxed. Initially midwives discussed how labouring and birthing in water empowered women. Jacquie noted ‘*I feel women have more control*’. Anna agreed suggesting she thought it was to do with power stating ‘*The woman holds more of the power in labour*’. Noreen continued the discussion ‘*the thing is society brings up pictures of women with somebody doing it* [the birth] *for them, there is a cultural thing of having somebody delivering the baby whilst* [with water] *there is themselves and their body*’. Bonnie reflected on Noreen’s comments suggesting water promoted a change in the woman’s demeanour ‘*You can see the change in the woman’s face and in her body when she gets in the water, it’s nice and relaxed*’. Beth agreed water ‘*Promotes the environment to be quiet and peaceful*’. Jacquie thought this may be because ‘*The space between contractions is very different from a land birth, they are very much more focused on their breathing and calmer*’. Whilst Noreen shared how a woman’s relaxed state affected the care she gave ‘*You know it’s all relaxed and you can concentrate more on the signs, the natural signs of a woman giving birth*’*.* Sophie agreed ‘*It’s so calming for the women. I think it relaxes them which then relaxes us*’.

### Undisturbed space

The theme ‘undisturbed space’, described how water creates an undisturbed space where access to the woman is mediated by the water. Jasmine noted that ‘*If you’re in the bath people knock and they stay out, they leave you alone. As far as society is concerned, it’s not acceptable to walk into the room when someone’s in the bath. If someone’s in lithotomy, fine*’. Kerry reflected it also had an impact on how safe the woman felt. ‘*Especially for the women who have a sexual abuse history, they feel safer in the water, they feel like you can’t get at them*’. The topic of safety led to a discussion around privacy with Olivia commenting that ‘*It’s* [‘water] *their ‘own space and you have to really reach into their space, rather than them being poked and prodded* [with a land birth]’. Dorothy agreed stating ‘*It’s more undisturbed*’. Kerry continued ‘*Even though you can see beneath the water and everything, I think for them it just feels, more private under the water*’. Kate reflected on her experiences by recounting a scenario ‘*A woman that came back to the waterbirth study day and spoke about when she got in* [the pool] *there was a real sense of privacy, even though she had nothing on, the water was like a veil*’. Baily also remarked on how the ‘dynamics’ of a labour in water effects the partner ‘*I get a sense they quite like it too, because they are able to just sit and observe and hold that silent still place…my experience is that even men feel quite comfortable in that space*’.

### The challenges midwives experienced with waterbirth

Analysis of the focus group transcripts exploring the question ‘are there any issues with waterbirth’ revealed that issues highlighted by the midwives were perceived as challenges. Two themes were identified: learning through reflection and facilities required to support waterbirth.

### Learning through reflection

The theme ‘learning through reflection’ illustrates how midwives learnt by documenting and then reflecting on the clinical challenges encountered during their day to day clinical practice around water immersion for labour and birth. Kerry shared ‘*I didn’t used to but since we’ve been doing group practice… when you look at your records you can see most of them are waterbirths*’. Olivia continued ‘*I don’t remember all of the waterbirths…I’ve got a little book that I just pop them in*’. Kate reflected on her colleagues comments sharing she did not keep records of each waterbirth and that her confidence caring for women in water ‘*took a long time. I’ve probably done, I don’t know over 150 now*’. Kate went on to explain why ‘*You had to flex the head and then move the hand and then sweep the perineum, it was really hands on. But that’s how we were taught. So to move to totally hands off* [waterbirth] *where you’re not even poised is challenging*’. Olivia agreed with Kate’s sentiments describing a waterbirth scenario where ‘*I remember taking over from somebody else and it was a hypno-birth and so there was no talking…it was a good learning experience*’.

To illustrate, the topic of learning through clinical experience led to a discussion around placental cord snapping. Bonnie shared ‘*I’ve had a few cord snaps now. Like quite a few issues, but it hasn’t changed my feeling of how to perform waterbirth because I know it’s going to be fine and we just deal with it as it comes*’. Kerry empathised, supporting Bonnie by acknowledging ‘*I think a lot of midwives get anxious even though they may pretend they don’t get anxious about waterbirths. They want to get the baby out as fast as possible. But I think if you make them* [the women] *aware you don’t just yank it* [the baby up]…*you need to check how long it* [the cord] *is before you can go yanking’*.

### Facilities required to support waterbirth

The theme ‘facilities required to support waterbirth’ related to ensuring waterbirth facilities were suitable, available and accessible for women and identified challenges relating to the provision of infrastructure around waterbirth. Jasmine stated:

*If we want this option* [waterbirth] *open for all women then we need to provide the facilities for that to happen. I have an issue with it being inequitable at the moment. The Birth Centre has the birth pool and blow up pools that are free of charge whilst clients* [women] *in the main hospital and CMP have to pay and hire their own…how come one group of clients under the same public system get it for nothing and the other group have to pay?*

Sophie was also concerned by the rollout of waterbirths to the main hospital but her frustration was around the referral process. ‘*When waterbith was approved in the main hospital…I had a patient come over and say ‘I want a waterbirth but they* [the main hospital] *won’t facilitate one for me over there and they’ve told me to come to the Birth Centre and I was quite surprised*’. Whilst Kate’s sentiments concerned the content of the waterbirth guidelines. ‘*When it* [the waterbirth guideline] *was first developed we didn’t have telemetry and now we do. So I think waterbirth telemetry needs to be incorporated into the guideline’*. Other midwives did not appear sure of how often in-service needed to be provided in relation to emergency management, pool evacuation and assessment of blood loss. There was debate between midwives in relation to how often these drills should be performed. Dorothy confirmed ‘*In the CMP we have to do like a quiz, you know we put the blood in the water every six months and estimate it*’. Whilst Jacquie confirmed ‘*We do up a calendar* [of available professional development sessions]’ and it was up to individuals to ensure their development was up to date.

## Discussion

This mixed methods study enabled us to explore midwives’ experience of their education, knowledge and practice around immersion in water for labour or birth in WA. Quantitative analysis found the majority of midwives felt equipped following waterbirth training to facilitate labour and birth in water, with scenario responses indicating midwives were practicing according to the WA state-wide guidance. Additionally, midwives were confident and enjoyed caring for women who used water immersion. Qualitative exploration of what midwives enjoyed about caring for women who used water immersion for labour and/or birth revealed three distinctive themes: instinctive birthing; woman-centred atmosphere; and undisturbed space. Whilst exploration of the challenges experienced with waterbirth revealed two themes: learning through reflection and facilities required to support waterbirth. Our discussion will focus on what waterbirth offers midwives.

Labouring and birthing in water is centred around the philosophy that pregnancy and birth are normal life events [19]. The importance of sustaining a waterbirth culture highlighted by these WA midwives aligns with the belief that maintaining low risk birth cultures is essential to meet the needs of healthy, low risk women through recognition and respect of midwives’ contribution [22]. Midwives in this study were experts in their field, who had been qualified for a mean of 13 years and five months and facilitating waterbirth for a mean of eight years and nine months; similar to other research [6]. During the study it was agreed that immersion in water for labour and birth could be facilitated in the tertiary Labour and Birth Suite. We suggest this expertise will be integral in relation to supporting midwives in the tertiary Labour and Birth Suite to become skilled waterbirth practitioners. Indeed, an action research study introducing a problem solving waterbirth workshop with UK midwives and their co-ordinators positively affected change in waterbirth practice and was recognised for its potential shift toward normalising low risk midwifery care [6].

Midwives are guided by the International Confederation of Midwives (ICM) Position Statement on ‘keeping birth normal’ [23] which asserts that midwives are advocates and experts in low risk childbirth. The ICM acknowledges that ‘women should have access to midwifery-led care, one-to-one support, including the choice of a home birth and immersion in water’ [23] which aligns with the international recommended pathway towards evidence based respectful maternity care [24]. Utilising immersion for labour and/or birth provides midwives with an opportunity to facilitate this experience for women.

The theme of ‘learning through reflection’ articulated by the midwives supports the ICM Philosophy of Midwifery Care [25] ensuring competent midwifery care is informed and guided by continuous education. The association between workplace learning and competence was confirmed in a Japanese study with nurse/midwives who related learning through reflection to their self-reported competence [26]. Differences were noted based upon level of experience whereby learning from feedback and training were associated with competence for more experienced clinicians compared to learning through practice and from others for self-reported competence for those with less experience [26]. Fittingly, the Australian national competency standards for the midwife [27] present domains around the provision of woman-centred care, with one domain suitably entitled ‘reflective and ethical practice’. Midwives in this study reinforce the relevance of this domain in their practice as both the clinical scenarios and focus group findings illustrated they valued having the ability and skills to analyse and reflect in, on and about practice to ultimately maintain clinical competence and confidence. In short, when care is provided by midwives who are educated [28, 29], regulated [21, 30] and provide respectful evidence based care [24], the outcomes are improved for women and their infants [1, 24, 28, 29]. The midwives in this study adhered to these principles empowering women to realise their potential to birth, though the medium of water.

### Strengths and limitations

Although the quantitative methods employed provided limited scope to explore the wide range of experiences midwives in our study encountered caring for women who laboured and/or birthed in water, they did provide the research team with an objective starting point for further exploration of specific aspects of the questionnaire [21, 30]. For example, utilising a question for the focus groups gleaned from a phase one question asking midwives to score their enjoyment facilitating immersion in water for labour and birth, gave us the opportunity to contextualise what they enjoyed; providing a connection between the quantitative and qualitative components that could not be answered by mono-methods alone. By utilising both numbers and words to explore this topic [14, 15], the qualitative and quantitative components became cohesively integrated, producing research findings around midwives enjoyment which were greater than the sum of individual parts of the research [31]. This approach exposed the importance of instinctive birthing; woman-centred atmosphere; and undisturbed space.

Midwives in this study were self-selected from the MGP and CMP midwives based within the sole tertiary public maternity hospital in WA. Providing midwives with a website link to the WA state-wide waterbirth guidelines may have influenced their responses. This was a self-assessment of competence which is a subjective aptitude. The research would have been strengthened by comparing the midwives responses to their actions. Participating midwives may have been motivated and confident in their waterbirth practice. The sample of midwives included in phase one was small and may not be representative of all midwives who provide care for women who labour and/or birth in water. We acknowledge these factors could have had an impact in relation to the findings and should be considered when interpreting transferability of the findings to other settings.

## Conclusion

This research contributes to the growing knowledge base examining midwives' education, knowledge and practice around immersion in water for labour or birth. It also highlights the importance of exploring what immersion in water for labour and birth offers midwives, as this research suggests they are an integral component in relation to supporting and sustaining a waterbirth culture. Midwives in this WA study were both competent and confident and enjoyed caring for women who used water immersion. Perhaps this was because the medium of water not only empowered women to realise their potential, but also themselves.

## Additional files

Additional file 1: Midwives satisfaction with waterbirth questionnaire. (PDF 184 kb)

Additional file 2: Focus group questions. (DOCX 12 kb)

## Abbreviations

CMP: Community Midwifery Program
FBC: Family Birth Centre
ICM: International Confederation of Midwives
KEMH: King Edward Memorial Hospital
MGP: Midwifery Group Practice
UK: United Kindgom
WA: Western Australia
