# Supplementary material for: Midwives’ experience of their education, knowledge and practice around immersion in water for labour or birth
Source: BMC Pregnancy Childbirth. 2018 Jun 19;18:249. doi: 10.1186/s12884-018-1823-0 (PMC6008918; doi:10.1186/s12884-018-1823-0)
Supplement: Supplementary file 1 — Midwives satisfaction with waterbirth questionnaire. (PDF 184 kb) [file 12884_2018_1823_MOESM1_ESM.pdf]

Midwives’ perception of their education, knowledge and practice around immersion in water for labour or birth

About you

1. How many years have you been working as a midwife?

Years_____ Months_____

2. How many years have you been facilitating immersion in water for labour or birth?

Years_____ Months_____

3. Where is your main place of work?

☐ MGP ☐ CMP

Your waterbirth education and training

4. What training did you undertake in preparation to facilitate immersion in water for labour or birth (Please tick all that apply)

☐ King Edward Memorial Hospital E-learning

☐Received and read ‘WA Clinical Guidelines for women requesting immersion in water for pain management during labour and/or birth

☐ King Edward Memorial Hospital study day

☐Observed waterbirth being facilitated by midwife competent in waterbirth

☐Facilitated waterbirth supervised by midwife competent in waterbirth

☐Other (Please describe)____

5. Following your education and training, did you feel well equipped to facilitate immersion in water for labour or birth?

☐ Yes ☐ No

Please describe?____

____

Your practice and waterbirth

6.How many waterbirths did you facilitate before you felt confident in waterbirth?________

7. Please describe two factors you would discuss with a woman in her scheduled antenatal appointments in relation to immersion in water for labour or birth?

Factor 1: ____

Factor 2: ____

Please respond to the questions below by selecting the number that best reflects your confidence/enjoyment.

8. How **confident** do you feel caring for a woman in water during the 1st stage of labour?

Not confident	0	1	2	3	4	5	6	7	8	9	10	Very confident

9. How **confident** do you feel caring for a woman in water during the 2nd stage of labour? (Please circle response)

Not confident	0	1	2	3	4	5	6	7	8	9	10	Very confident

10. How **confident** do you feel caring for a woman in water during the 3rd stage of labour? (Please circle response)

Not confident	0	1	2	3	4	5	6	7	8	9	10	Very confident

11. How **confident** do you feel using the emergency evacuation system to get a woman out of the bath (Please circle response)

Not confident	0	1	2	3	4	5	6	7	8	9	10	Very confident

12. I **enjoy** facilitating immersion in water for labour (Please circle response)

Do not enjoy	0	1	2	3	4	5	6	7	8	9	10	Enjoy

13. I **enjoy** facilitating immersion in water for birth (Please circle response)

Do not enjoy	0	1	2	3	4	5	6	7	8	9	10	Enjoy

14. I actively promote the use of water for labour to all eligible women

☐ Yes ☐ No

Please describe?____

____

15. I actively promote the use of water for birth to all eligible women

☐ Yes ☐ No

Please describe?____

____

Scenarios

The following scenarios are based on the Department of Health West Australian 'Water Birth Clinical Guidelines' (http://www.health.wa.gov.au/circularsnew/attachments/726.pdf). Please feel free to access these evidence based guidelines in responding to the scenarios below.

Please read the following scenarios and respond to the questions in the space below each section.

Scenario 1

Anna is a 30 year old, G2P1, attending her 34 week antenatal appointment. In her last labour she utilised epidural analgesia for pain relief. She had an SVD, of a healthy baby boy weighing 3.8kg.

Her current pregnancy is low risk. She signed the 'Agreement for use of water for labour and/or birth' form at her last antenatal visit. She is hoping to birth vaginally without pharmacological pain relief. Today, Anna tells you she is worried about giving birth without pharmacological pain relief and concerned that using the bath may lengthen her labour.

How would you advise her OR what would you discuss with her to allay her concerns?

Scenario 2

Anna is now 40 weeks and presented in spontaneous labour, membranes intact, contracting 4:10. She has been in the bath for 2 hours and states she is ‘feeling hot’. When you check her temperature it is 37.6.

What would your initial plan be to ensure fetal and maternal wellbeing?

Scenario 3

Anna is back in the bath and has been pushing for 40 minutes. She is about to birth. Only Anna, her partner and yourself are in the room.

How would you prepare for and facilitate the birth*?*

Scenario 4

Anna gives birth in water. Her healthy, baby girl is now on her chest. Anna has requested a physiological 3rd stage in the bath. Ten minutes after birth the placenta has not been delivered and Anna appears to be bleeding.

What would your management be?

Thank you for your time and contribution

If you would be willing to attend a focus group with your MGP/CMP colleagues to discuss your experiences around water for labour or birth, please tear off this slip and place it in the box in the FBC tea room. Discussion may include feedback around your training, mentorship and other issues you feel are important in relation to this topic.

First name ……………………………Work mobile...
