# Supplementary material for: Midwives’ experience of their education, knowledge and practice around immersion in water for labour or birth
Source: BMC Pregnancy Childbirth. 2018 Jun 19;18:249. doi: 10.1186/s12884-018-1823-0 (PMC6008918; doi:10.1186/s12884-018-1823-0)
Supplement: Supplementary file 2 — Focus group questions. (DOCX 12 kb) [file 12884_2018_1823_MOESM2_ESM.docx]

**Focus group questions**

**Question 1**

What contributes to your enjoyment of waterbirth?

**Prompt 1**

How does waterbirth contribute towards natural birth?

**Prompt 2**

How does waterbirth promote women’s choice?

**Question 2**

Are there any issues with waterbirth?

**Prompt 1**

Which stage of a waterbirth labour do you find most challenging?
